# Warshaw Technique in Laparoscopic Spleen-Preserving Distal Pancreatectomy: Surgical Strategy and Late Outcomes of Splenic Preservation

**DOI:** 10.1155/2019/4074369

**Published:** 2019-06-17

**Authors:** Lei Wang, Dong Wu, Yu-gang Cheng, Jian-wei Xu, Hai-bo Chu, Guang-yong Zhang, San-yuan Hu, Han-xiang Zhan

**Affiliations:** ^1^Department of General Surgery, Qilu Hospital, Shandong University, Jinan, Shandong Province, 250012, China; ^2^Department of General Surgery, 89th Hospital of the People's Liberation Army, Weifang, Shandong Province, 261000, China

## Abstract

Laparoscopic spleen-preserving distal pancreatectomy (LSPDP) can be accomplished with either the preservation or the resection of splenic vessels; the latter is also known as Warshaw technique. Our study is designed to investigate the operation selection strategy when proceeding LSPDP and to evaluate the long-term outcomes of patients undergoing Warshaw surgery. The medical records and follow-up data of patients who underwent LSPDP in Qilu Hospital, Shandong University, were reviewed retrospectively. A total of thirty-five patients were involved in this study, including 17 cases of patients who were treated with Warshaw procedure (WT) while the other 18 cases had splenic vessels preserved (SVP). Compared with the SVP group, the operative time and intraoperative blood loss in WT group were improved significantly. The incidence of early postoperative splenic infarction was higher in WT group. However, there was no report of splenic abscess or second operation. Follow-up data confirmed that there was no significant difference in spleen phagocytosis and immune function compared with normal healthy population. Our study confirms that LSPDP-Warshaw procedure is a safe and efficient treatment for the benign or low grade malignant tumors in distal pancreas in selected patients. The long-term spleen function is normal after Warshaw procedure. Preoperative assessment and intraoperative exploration are recommended for the selection of operation approaches.

## 1. Introduction

Pancreatic surgery is considered as one of the most challenging surgical procedures in abdominal surgery [1]. Some anatomic disadvantages, such as complex proximity to the major vasculature and retroperitoneal location, have once restrained the application of laparoscopic techniques in pancreas area [2]. With the advances in surgical skills and laparoscopy instruments, laparoscopic distal pancreatectomy (LDP) has been widely utilized for the treatment of benign lesions or low-grade malignancies in the body and tail of the pancreas [3]. For the sake of technical simplicity, concomitant splenectomy was routinely performed at the initial stage of LDP. Along with a better understanding of the immunologic role of spleen through these years, researchers confirmed that the removal of spleen will put the patients at a potential risk of severe complications, such as overwhelming postsplenectomy infection (OPSI) and postoperative thrombocytosis [4–6]. At current clinical practice, surgeons tend to preserve the spleen whenever possible. Not only did patients benefit from minimal invasive and enhanced recovery after surgery, their long-term life quality was also significantly improved after laparoscopic spleen-preserving distal pancreatectomy (LSPDP) [7].

Spleen-preserving distal pancreatectomy (SPDP) can be accomplished with either resection or preservation of the spleen vessels. SPDP with ligation of splenic artery and vein was first reported by Warshaw et al. from Massachusetts General Hospital in 1988 [8]. And this surgical procedure was later named after Doctor Warshaw. In 1996, Kimura et al. from Yamagata University described the splenic vessels preservation (SVP) in SPDP for the first time [9]. When performing SVP, an unavoidable technical challenge would be the diversion of numerous of short branches from the splenic vein and artery spreading to pancreatic body and tail, which requires special cautions and is also time consuming. In recent years, the Warshaw procedure has gained the favor of many laparoscopic surgeons considering its relative convenience of operation. Meanwhile, there were also previous studies which revealed that Warshaw technique was associated with higher risk of splenic infarction and gastric varices as well as a theoretical potential of gastrointestinal bleeding during long-term follow-up [10, 11].

Studies comparing short-term outcomes between Warshaw and SVP showed different results [12–14]. Of note, little attention has been paid to the long-term outcomes of the Warshaw technique, especially for the immune functions of the preserved spleens. Here in this study, we assessed the postoperative outcome of LSPDP patients in our institute. Long-term splenic function was highlighted. By doing so, we sought to clarify the indication of LSPDP Warshaw procedure, to help surgeons improve intraoperative decision-making strategies.

## 2. Materials and Methods

### 2.1. Study Design

A total of 35 patients, including 9 males and 26 females, were recruited consecutively from January 2015 to April 2018. These patients were diagnosed with benign or low-grade malignant pancreatic tumors and underwent LSPDP in Qilu Hospital of Shandong University. For all 35 cases, imaging data were carefully evaluated by the whole surgical group before surgery. Special attention was paid to the anatomical relationship between the tumor and splenic vessels. Operation methods were decided by operator based on the possibility of the splenic vessels dissected from the tumors. Of those, 17 patients underwent Warshaw procedures (WT group) and the other 18 patients had the splenic vessels preservation during LSPDP (SVP group). We also performed a subgroup analysis within the WT group. Based on a careful screening of the operative documents, we identified 8 WT patients who initially intended to accept SVP procedure. However, inevitable conversions to WT procedure were performed in these 8 cases owing to adhesions of the tumor to the splenic vessels (Trans-WT group). The other 9 WT patients were defined as planned straight-WT group.

All patients routinely underwent enhanced abdominal computed tomography (CT) scan at one week and three months after surgery. Five of 17 patients in Warshaw group received 99m-Tc sulfur colloid spleen scan examination at their two-year revisit. Blood tests including platelet count (before surgery as well as Day 1, Day 3, and Day 7 after surgery), immune globulin, and lymphocyte subsets (one-year after surgery) were performed in the clinical laboratory of Qilu Hospital for all groups. One-year-after-surgery serum Tuftsin levels were also evaluated using Tuftsin ELISA kit (Shanghai Run Yu Biotechnology Co., China).

Medical records were reviewed retrospectively for demographic details, operation procedures, postoperative parameters, and follow-up data. Special attention has been paid to the splenic function. Our study was approved by the local ethics committee. All of the patients signed the written informed consent for the scientific research.

### 2.2. Surgical Techniques

Surgical procedures for LSPDP included transection of the splenic artery and vein (Warshaw technique) and splenic vessels preservation (SVP technique).

#### 2.2.1. Warshaw Technique

The patient was placed in supine reverse Trendelenburg position under general anesthesia. We placed the first 10 mm trocar in the umbilicus for the location of a 30° electrolaparoscope (KARL STORZ, Tuttlingen, Germany) and the intra-abdominal pressure was set at 12 mmHg. Then one 12-mm and one 5-mm trocars were placed in the right upper quadrant under direct vision for the surgeon, followed by two 5-mm trocars in the left upper quadrant for the assistant. The surgical procedure can be described as follows. We divided the greater omentum below the gastroepiploic arch to get access to the bursa omentalis. Gastrocolic ligaments were also dissected using the ultrasonic scalpel to reveal the pancreatic lesion. Intraoperative laparoscopic ultrasound was used to locate the tumor and to predetermine the surgical resection line when necessary. Close attention was paid to protect the short gastric vessels and left gastroepiploic vessels. Then the inferior border of pancreas was liberated from the transverse colon by blunt dissection, revealing the splenic vein. The pancreatic body was further separated from the retroperitoneum toward the upper border of the pancreas until the splenic artery was visualized and the retropancreas tunnel was established. A linear endoscopic stapler was inserted through the tunnel. Splenic vessels and pancreatic parenchyma were compressed and cut with the stapler. The distal pancreas was then gently lifted and ultrasonic scalpel was used to separate the loose tissue in the retroperitoneal space until the level of splenic hilum. Distal sides of the splenic artery and vein were sectioned using the endoscopic stapler.

**Figure 1:**
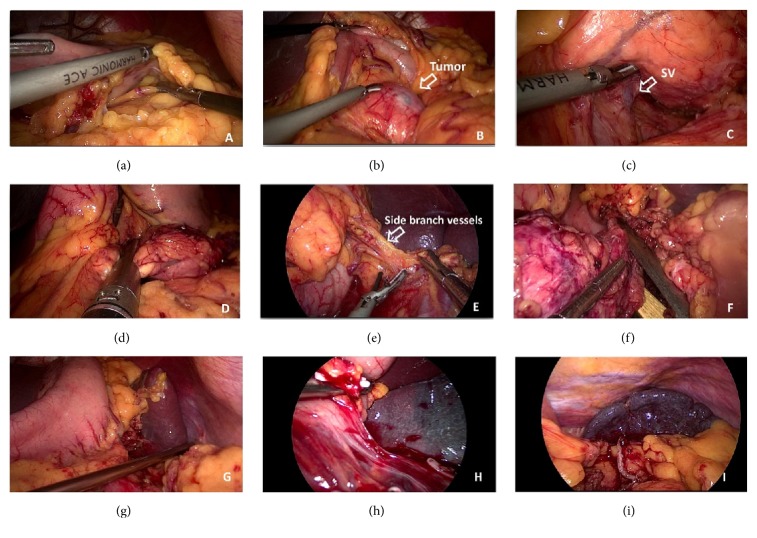
Illustrations for key steps in Warshaw procedure. (a) Get access to the bursa omentalis. (b) Reveal the pancreatic lesion. (c) Establish the retropancreas tunnel. (d) Splenic vessels and pancreatic parenchyma compressed and cut with the stapler. (e) Pay attention to protecting the side branch vessels. (f) Distal sides of the splenic vessels were sectioned using the endoscopic stapler. (g/h/i) Representative pictures showing different ischemic states of the spleen.

All bleeding points were stopped after careful inspection. A drainage tube was placed at the pancreatic remnant. The specimen was finally removed in an endoscopic bag through the extended umbilical incision. The illustrations for key steps in Warshaw procedure were summarized in Figure 1.

#### 2.2.2. SVP Technique

The patients' position and the trocar placement were the same as in the Warshaw procedure. After the exploration of the abdominal cavity and the revealing of the pancreatic lesion, the inferior pancreatic border was separated to expose the splenic vein on the lower edge of the pancreas. The splenic artery was dissected and encircled with tape. After careful separation of splenic vessels from the pancreas, an endoscopic stapler was inserted between the vessels and parenchyma. After compressing and cutting off the pancreatic parenchyma, the distal pancreas was pulled up ventrally to allow for the dissection along the splenic vessels to the splenic hilum. The small vessel branches were occluded carefully using knot and tie ligation, clips, or ultrasonic scalpel. The specimen was extracted through the enlarged umbilicus incision.

### 2.3. Statistical Analysis

Categorical variables were presented as counts (percentages) and continuous variables are expressed as mean ± standard deviation. Statistical differences between groups were evaluated by the chi-square test and Fisher's exact test, Student's t-test, and paired t-test, respectively. Comparisons between quantitative variables were performed using unpaired Student's t-test or one-way analysis of variance (ANOVA), followed by the Tukey-Kramer post hoc test. Chi-square test and Fisher's exact test were used to compare categorical variables. Two-sided* P* values ≤ 0.05 were considered statistically significant. SPSS 20.0 (SPSS Inc., Chicago, IL, USA) was used for statistical analysis.

## 3. Results

### 3.1. Patient Characteristics

Thirty-five patients (26 females and 9 males) were involved in this study, with a mean age of 48.2 ± 14.4 years old. Seventeen patients underwent Warshaw procedures based on preoperative assessment that the splenic vessels could not be safely separated from the tumor. The other 18 patients (SVP group) had the splenic vessels preservation during LSPDP. We found that female patients made up the majority of the cases in either WT or SVP group. Most of the patients in both groups reported abdominal pain as the chief complaint. There was also no regularity in terms of tumor sites. We found significant differences in tumor size between WT and SVP groups. The mean tumor size in Warshaw patients was 4.1 ± 1.6 cm, which was much larger than that in the SVP patients (2.9 ± 1.6 cm) (p<0.01). When it comes to histological diagnosis, there were no significant differences between WT group and SVP group. The detailed information was described in Table 1.

### 3.2. Perioperative Comparison between WT Group and SVP Group

As illustrated in Table 2, operation time was significantly shorter in WT group than SVP group (185.4 ± 40.2 versus 226.4 ± 56.3 min, p=0.019). We could also expect less blood loss for patients who underwent Warshaw surgery (87.2 ± 47.4 versus 124.6 ± 56.3 ml, p=0.042). There were one case conversion (from laparoscopic surgery to open surgery) in WT group and two cases conversions in SVP group due to intraoperative massive bleeding. Patients recovered well after surgery. There was no 30-day mortality in both groups. One patient in WT group and three cases in SVP group had Grade B pancreatic fistula after surgery. There exists no statistically significant difference among these two groups regarding postoperative hospital stay and total medical expenses. Five patients (5/15, 33.3%, CT information unavailable in 2 cases) were diagnosed as postoperative splenic infarction through abdominal CT scanning in WT group. None of them aggravated to splenic abscesses and they needed no reoperation during follow-up. Meanwhile, there was no splenic infarction in SVP group after surgery. One patient in WT group presented with postoperative perigastric varies and was observed without any treatment, with no gastric hemorrhage 12 months after the operation.

### 3.3. Comparison between Straight-WT and Trans-WT

We also carried out a subgroup analysis for the WT group based on the initial surgical strategy. There were 8 patients who were designed to proceed with the SVP in the first place. However, a transition to Warshaw procedure was made during the operation after the intraoperative exploration and the attempt to preserve splenic vessels. The WT group were then divided into two groups, which were Straight-WT (n=9) and Trans-WT (n=8). Tumor size in Trans-WT groups seems to be bigger than Straight-WT, while there existed no statistical difference (p=0.156). It took remarkable less time in group of Straight-WT than that in the Trans-WT group (140.6 ± 60.7 versus 207.5 ± 43.2 min, p=0.021). Blood loss was also significantly decreased in the Straight-WT group (94.4 ± 63.3 versus 193.8±80.8 ml, p=0.012). No statistical difference was found in terms of histologic features and tumor site between the two groups. Detailed data were summarized in Table 3.

### 3.4. Long-Term Follow-Up of Warshaw Procedure

**Figure 2:**
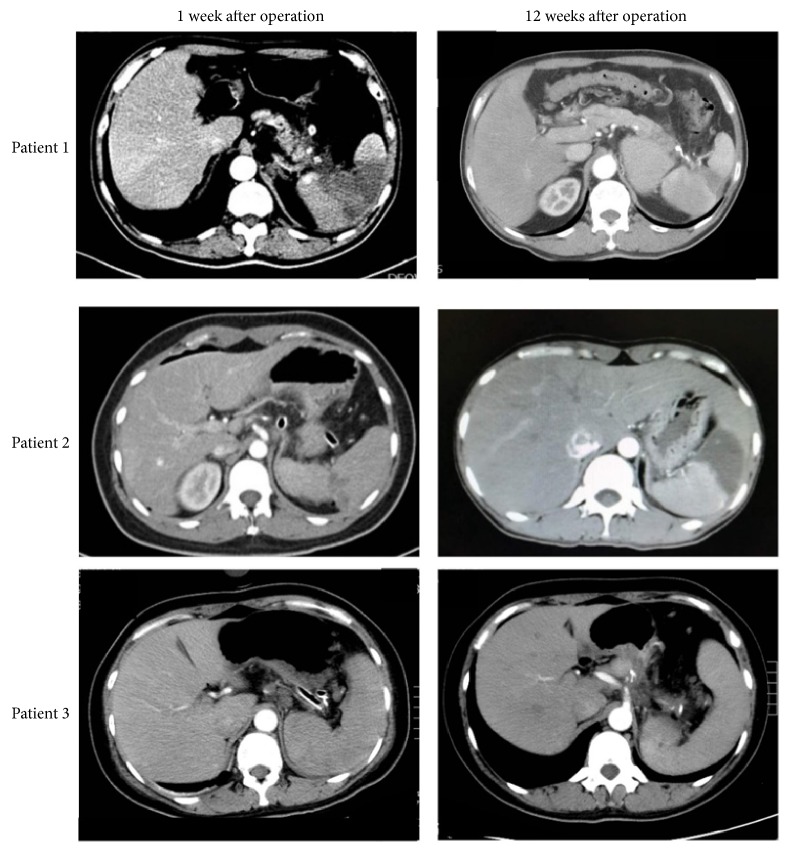
CT scanning images showing splenic infarction recovery during following up. Patient 1 had severe splenic infarction at 1 week after surgery. The infarction was obviously improved at week 12. The splenic infarction in patient 2 developed between the two follow-up visits. Patient 3 had only mild splenic infarction at week 1 and recovered completely after 12 weeks.

**Figure 3:**
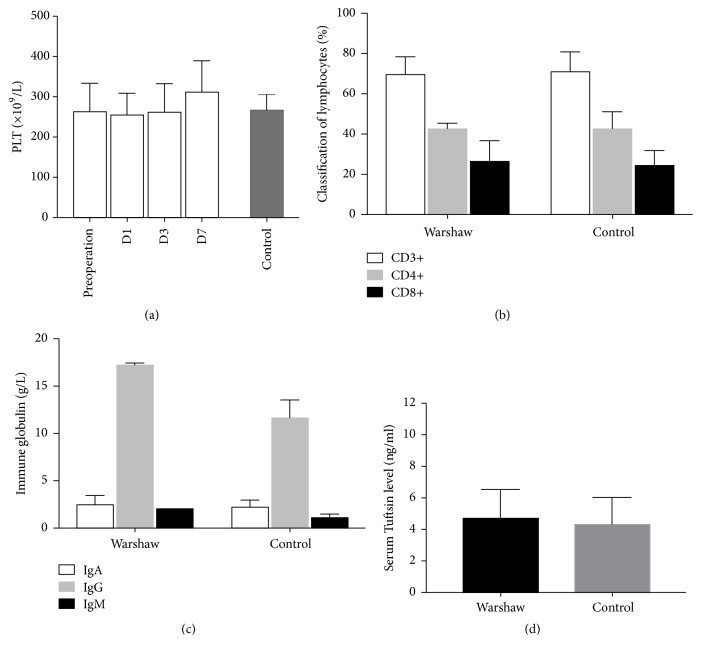
Splenic functions after Warshaw procedure were evaluated by platelet count (a), lymphocyte grouping (b), humoral immune globulins (c), and serum tuftsin level (d). There was no significant difference between patients who underwent Warshaw procedures and normal healthy population.

**Figure 4:**
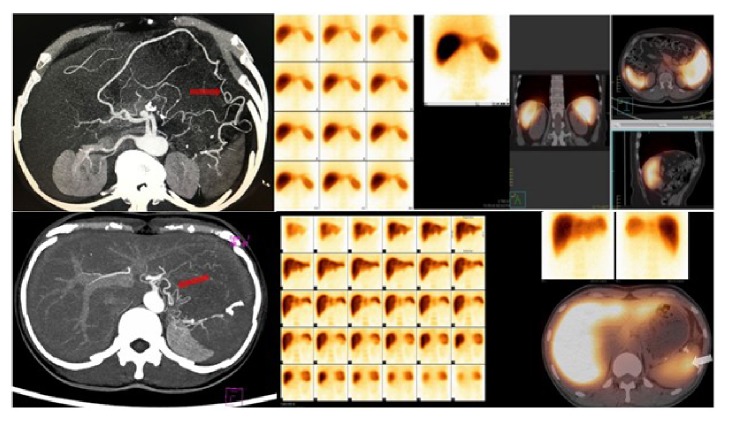
Representative images for 99m-Tc sulfur colloid liver and spleen scan after Warshaw procedure. The preserved spleen had normal phagocytic function (white arrow). Collateral circulations were also observed (red arrows).

Follow-up data were acquired from hospital records and supplemented with information collected from telephone interview with patients. CT scan results were unavailable in 2 patients. Five out of 15 patients (5/15, 33.3%) were diagnosed as postoperative splenic infarction through abdominal CT scanning in WT group.  Figure 2 showed the representative images of the dynamic changes during follow-up. All five patients received no specific treatment and no splenic abscess occurred. Blood tests were also applied to assess the splenic function after Warshaw procedure. Platelet counts were recorded before surgery and at Day 1, Day 3, and Day 7 after Warshaw procedure. Blood samples from healthy subjects were set as control group. No significant differences were observed between various times points (Figure 3(a)). Serum samples of one-year-follow-up point were also analyzed. There was no statistical difference between Warshaw group and health control when it comes to immune globulin and lymphocytes subsets (Figures 3(b) and 3(c)). Serum Tuftsin, a sensitive marker of splenic phagocyte function [20], remained at a normal level in patients after Warshaw surgery (Figure 3(d)). Five of 17 patients underwent 99m-Tc sulfur colloid spleen scan at their revisiting examination. All patients exhibit normal splenic function after surgery. Collateral circulations were also observed (Figure 4).

## 4. Discussion

In this retrospective study, we presented our initial experience with LSPDP in 35 consecutive patients during a 3-year period. Different from previous reported studies, we paid particular attention to the surgical strategy for the Warshaw procedure during LSPDP in this study. Of note, we also investigated the splenic complications and long-term functions of preserved spleens after Warshaw procedure. The results show that Warshaw procedure is safe and much faster and easier than SVP; postoperative pancreatic fistula rate and hospital stay are similar in Warshaw and SVP groups. Patients who underwent Warshaw procedure presented with more splenic infarction than SVP (33.3% VS 0%). However, these patients were observed without any treatment, and no splenic abscess occurred. More important, the phagocytic and immune functions of preserved spleen are normal, even in patients who had splenic infarction after Warshaw procedures. There were one patient in WT group and three patients in SVP group who developed Grade B pancreatic fistula after surgery. All these four patients recovered satisfactorily after adequate drainage. Warshaw procedure would not increase serious fistula rate after surgery, which has been established in previous studies [15, 21].

Professor Andrew Warshaw reported the first SPDP case series in 1988 [8]. Twenty-two of 25 consecutive patients were performed successfully; only 1 patient had late splenic abscess. In 1996, Wataru Kimura reported the SPDP with splenic vessels preservation (SVP) [9, 22]. Numbers of previous retrospective studies and meta-analysis have compared the advantages and disadvantages between SVP and Warshaw technique (WT). The results showed that SVP had lower splenic infarction, longer operation time, and more intraoperative blood loss, while WT had association with shorter operation time, less blood loss, more splenic infarction, and more perigastric varices [14, 15, 21, 23–27]. Although Warshaw procedure has many advantages as mentioned before, surgeons were always afraid of the splenic preservation related complications, such as splenic infarction and perigastric varices. The key issue for surgeons is to identify ideal candidates and then perform Warshaw procedures on these patients. In addition, functional situation of the preserved spleen in patients who received Warshaw procedures still remains unclear. To our knowledge, there are few studies that focus on the late outcomes of splenic preservation after WT procedure [17, 24, 28].

**Figure 5:**
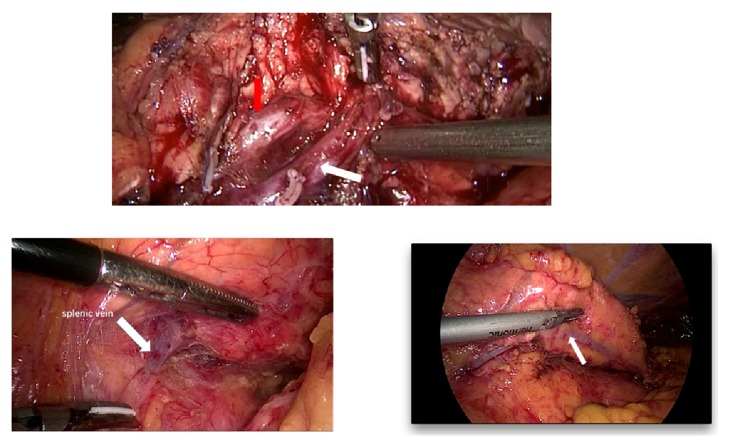
Intraoperative images showing the embedding of splenic artery (red arrows) and splenic vein (white arrows) in the sulcus of pancreatic parenchyma.

The key factors for splenic vessels preservation during distal pancreatectomy include the following: (1) tumor location (close to the splenic hilum or not); (2) splenic anatomy: embedded in pancreatic gland or not; (3) local inflammation presentation; (4) tumor invasion; (5) surgeon's experience [15, 16, 29, 30]. If the tumor locates in the tail of pancreas nearby the splenic hilum, dissection of the tumor and tail of pancreas from splenic vessels may be difficult, especially for the large tumor size (>5cm), and the situation may be worse when the local inflammation presented with or without history of acute pancreatitis. An important surgical procedure during SVP is dissection of splenic artery and vein from pancreatic parenchyma. Based on the previous literature [31] and our own experience, splenic vessels anatomy varies between different patients. The most common type is splenic artery (SA) curved and runs superior to the pancreas, while splenic artery in some patients passed relatively straight to the dorsal side of the pancreas; both SA and splenic vein (SV) are embedded in the sulcus of pancreatic parenchyma (Figure 5). For the latter type, dissection and ligation of small branches of splenic vessels may be difficult, SV can be easily injured, and unexpected massive bleeding may occur. Besides this, SVP is an especially time-consuming procedure in these patients, which increases pneumoperitoneum related complications, especially in elderly patients. For the intraoperative appearance of malignant tumor invasion to the splenic vessels or spleen, distal pancreatectomy and splenectomy are recommended instead of SPDP [5, 32]. We also compared the clinical data between patients who received straight WT and patients who underwent SVP transfer to WT. As in our study, eight patients were transmitted to Warshaw procedure after the attempt of splenic vessels preservation. After careful screening of the operation records, the main reason for transition can be summarized as follows: tumor sizes in two cases were relatively larger (5.2cm & 6.3cm); frequent bleeding due to local inflammation happened in four cases when transecting the vessels; the tumors in the other two cases were found too close to splenic hilum case after the intraoperative exploration. Shorter operation time and less blood loss were observed in straight WT group. Based on the results and our experience, straight WT procedure is recommended and SVP should not be applied for these patients: (1) tumor size >5cm and located close to the splenic hilum; (2) SA and SV were embedded in pancreatic gland; (3) local inflammation is present; (4) for fat, old people, long distance dissection of pancreatic gland is required (>5cm).

Splenic infarction and potential necrosis or abscess is one of the most common complications after Warshaw procedure and also a key issue for surgeon to worry about [10, 29]. 33.3% of patients (5/15) had partial splenic infarction in our study, which was similar to previous reported studies, but these 5 patients in our group were observed without any treatment, with no splenic abscess and splenectomy. Andrew Warshaw and his colleagues reported the 23 years' surgical experience of WT, and a total of 158 patients were included, and 1.9% (3/159) of patients underwent reoperations due to splenic infarction. During the long follow-up process, localized splenic malperfusion or infarction was confirmed in 23% of these 158 patients [17]. Rate of reoperation and splenectomy after WT ranges from 0% to 5.9% [10, 14–19, 33]. No splenectomy after WT has been reported since 2014, and this may be due to the improvement in the surgical skills. Development of perigastric varices is another great concern after ligation of splenic veins. Despite the high incidence, no postoperative upper gastrointestinal bleeding was reported during the long follow-up period (up to 24 years) [10, 14, 16, 17, 29]. The splenic complications following WT are summarized in Table 4.

How to perform Warshaw procedure safely? Protecting the left gastroepiploic artery, short gastric arteries, and posterior gastric artery to ensure the collateral circulation after SA interruption is the most important issue. Avoid dissection of the perisplenic ligaments, such as lienocolic and lienorenal ligament; this strategy can also help to prevent postoperative splenic ischemia. Another important tip for surgeons is to cut off the splenic vessels as far away from the splenic hilum with the complete resection of tail of pancreas, which can ensure the blood flow of left gastroepiploic artery. After pancreatic resection, surgeons should reexamine the spleen. Clear demarcation lines in the upper or lower pole of spleen indicate local infarction, and the spleen can be preserved if it is less than half the apparent surface [17]. If the color of spleen after pancreatic resection is red dark in some areas or burgundy, the spleen can also be preserved, but if the color is dark gray or black, splenectomy is recommended for these patients [17, 29].

Evaluation of the immune and phagocytic function of preserved spleens after WT procedure is a bright spot of our study. Serum Tuftsin, Serum IgA, IgG, IgM, and CD3^+^, CD4^+^, and CD8^+^ T cell subset counts were analyzed in these patients. The levels of serum Tuftsin, humoral immunity, and cellular immunity have no significant difference between patients who underwent WT and healthy control, which indicating a normal immune function of preserved spleen. Besides this, we also performed 99m-Tc sulfur colloid ECT scan on these patients; the results showed that preserved spleen presented with normal phagocytic function. These results confirmed the safety, possibility, and necessity of Warshaw procedure, which can give surgeons more clinical evidence to make decision during operation and also much more confidence and courage to perform this procedure.

Due to the retrospective design, inevitable selection bias existed in this study; these operations were performed by two different surgical groups, different surgical approaches, and perioperative management, which could influence the results. Beside this, data of our study was from a single center, and sample size was small. Multicenters, randomized, large samples study may be helpful to further elucidate the clinical value and surgical strategies of Warshaw procedure.

In conclusion, both laparoscopic Warshaw procedure and SVP are safe, feasible, and efficient, and Warshaw technique is much faster, easier, and safer. Splenic infarction and perigastric varices after Warshaw procedure are acceptable and usually can be observed without any treatment. Of note, preserved splenic function is normal during long-term follow-up process. For selected patients as mentioned before, Warshaw procedure is recommended instead of SVP. Most important, surgeons should adjust the surgical strategies during LSPDP flexibly according to the lesion features and actual situations.

## Figures and Tables

**Table 1:** Patients demographics.

Characteristics	WT (n=17)	SVP (n=18)	*p* value
Gender			NS
Male	2 (11.8)	7 (38.9)	
Female	15 (88.2)	11 (61.1)	
Age, M ± SD, Years	47.2 ± 15.1	49.1 ± 13.6	NS
<50	10 (58.8)	8 (44.4)	
≥50	7 (41.2)	10 (55.6)	
Symptom			NS
Without specific symptoms	5 (29.4)	4 (22.2)	
Abdominal pain/discomfort	10 (58.8)	12 (66.7)	
Others	2 (11.8)	2 (11.1)	
Tumor site			NS
Body	5 (29.4)	8 (44.5)	
Tail	5 (29.4)	6 (33.3)	
Body and tail	7 (41.2)	4 (22.2)	
Tumor size, M±SD, cm	4.1±1.6	2.9±1.6	p<0.01
< 5	10 (58.8)	14 (77.8)	
≥ 5	7 (41.2)	4 (22.2)	
Histologic features			NS
Solid pseudopapillary neoplasm	2 (11.8)	2 (11.1)	
Neuroendocrine neoplasm	6 (35.3)	7 (38.9)	
Serous cystadenoma	3 (17.6)	2 (11.1)	
Mucinous cystadenoma	4 (23.5)	4 (22.2)	
Intraductal papillary mucinous neoplasm	1 (5.9)	0 (0)	
Pancreatic cyst	1 (5.9)	3 (16.7)	

**Table 2:** Perioperative comparison between WT group and SVP group.

Features	WT	SVP	*p* value
Operative time (min)	185.4 ± 40.2	226.4 ± 56.3	0.019
Estimated blood loss (ml)	87.2 ± 47.4	124.6 ± 56.3	0.042
Conversion (case)	1	2	NS
Transfusion (case)	1	1	NS
Pancreatic fistula			NS
Biochemical leak	16	15	
Grade B&C	1	3	
Postoperative infarction of spleen	5	0	<0.001
Postoperative hospital stay	11.2 ± 5.2	11.4 ± 5.7	0.915
30-day mortality	0	0	NS
Total expenses	60538.1 ± 16086.0	58244.9 ± 15573.3	0.159

**Table 3:** Comparison between Straight-WT and Trans-WT.

Features	Straight-WT (n=9)	Trans-WT (n=8)	*p *value
Tumor size, M ± SD, cm	3.6 ± 1.8	4.7 ± 1.1	0.156
Tumor site			NS
Body	3 (33.3)	2 (25)	
Tail	3 (33.3)	2 (25)	
Body and tail	3 (33.3)	4 (50)	
Histologic features			NS
Solid pseudopapillary neoplasm	1 (11.1)	1 (12.5)	
Neuroendocrine neoplasm	1 (11.1)	5 (62.5)	
Serous cystadenoma	3 (33.3)	0 (0)	
Mucinous cystadenoma	3 (33.3)	1 (12.5)	
Intraductal papillary mucinous neoplasm	1 (11.1)	0 (0)	
Pancreatic pseudocyst	0 (0)	1 (12.5)	
Operative time (min)	140.6 ± 60.7	207.5 ± 43.2	0.021
Estimated blood loss (ml)	94.4 ± 63.3	193.8 ± 80.8	0.012

**Table 4:** Literature review for splenic complications following Warshaw procedure.

Author	Published year	Follow-up Period (month)	Sample	Number of splenic infarctions	Splenic infarction%	Number of splenectomies	Reoperation%	Number of perigastric varices	Perigastric varices%
Kim et al. [10]	2016	35	122	66	54.2	0	0	25	20.5
Matsushima et al. [14]	2014	45	17	4	24	0	0	2	12
Jean-Philippe et al. [15]	2013	-	85	9	10.5	4	5.9	-	-
Nakamura et al. [16]	2016	19	6	3	50	0	0	2	33
Ferrone et al. [17]	2011	32.4	158	15	23	3	1.9	16	25
Fernandez-Cruz et al. [18]	2007	38	34	7	20	0	0	-	-
Tien et al. [19]	2010	45.3	37	0	0	0	0	11	29.7

## Data Availability

The figures and tables data used to support the findings of this study are included within the article.
